# Supplementary material for: Clonal Myeloid Dysplasia Following CAR T-Cell Therapy: Chicken or the Egg?
Source: Cancers (Basel). 2023 Jul 3;15(13):3471. doi: 10.3390/cancers15133471 (PMC10341314; doi:10.3390/cancers15133471)
Supplement: Supplementary file 1 [file cancers-15-03471-s001.zip › cancers-2463346-supplementary.pdf]

Supplementary data

The list of genes included in the Archer® VariantPlex® panel:

Genes targeted for point mutations and indels: ABL1 ANKRD26 ASXL1 ATRX BCOR BCORL1 BRAF BTK CALR CBL CBLB CBLC CCND2 CDKN2A CEBPA CSF3R CUX1 CXCR4 DCK DDX41 DNMT3A ETNK1 ETV6 EZH2 FBXW7 FLT3 GATA1 GATA2 GNAS HRAS HX15 IDH1 IDH2 IKZF1 JAK2 JAK3 KDM6A KIT KMT2A KRAS LUC7L2 MAP2K1 MPL MYC MYD88 NF1 NOTCH1 NPM1 NRAS PDGFRA PHF6 PPM1D PTEN PTPN11 RAD21 RBBP6 RUNX1 SETBP1 SF3B1 SH2B3 SLC29A1 SMC1A SMC3 SRSF2 STAG2 STAT3 TET2 TP53 U2AF1 U2AF2 WT1 XPO1 ZRSR2

Genes targeted for CNV detection: ASXL1 FLT3 CDC25C TET2 BCOR IKZF1 U2AF1 TP53 CBL KDM6A RPS14 U2AF1 CUX1 LUC7L2 MYC WT1 ETV6 NF1 RAD21 ZRSR2 EZH2 CDKN2 RUNX1
